# Familial Resemblance of Borderline Personality Disorder Features: Genetic or Cultural Transmission?

**DOI:** 10.1371/journal.pone.0005334

**Published:** 2009-04-24

**Authors:** Marijn A. Distel, Irene Rebollo-Mesa, Gonneke Willemsen, Catherine A. Derom, Timothy J. Trull, Nicholas G. Martin, Dorret I. Boomsma

**Affiliations:** 1 Department of Biological Psychology, VU University Amsterdam, Amsterdam, The Netherlands; 2 Department of Human Genetics, Katholieke Universiteit Leuven, Leuven, Belgium; 3 Department of Psychological Sciences, University of Missouri-Columbia, Columbia, Missouri, United States of America; 4 Queensland Institute of Medical Research, Brisbane, Australia; Erasmus MC, Netherlands

## Abstract

Borderline personality disorder is a severe personality disorder for which genetic research has been limited to family studies and classical twin studies. These studies indicate that genetic effects explain 35 to 45% of the variance in borderline personality disorder and borderline personality features. However, effects of non-additive (dominance) genetic factors, non-random mating and cultural transmission have generally not been explored. In the present study an extended twin-family design was applied to self-report data of twins (N = 5,017) and their siblings (N = 1,266), parents (N = 3,064) and spouses (N = 939) from 4,015 families, to estimate the effects of additive and non-additive genetic and environmental factors, cultural transmission and non-random mating on individual differences in borderline personality features. Results showed that resemblance among biological relatives could completely be attributed to genetic effects. Variation in borderline personality features was explained by additive genetic (21%; 95% CI 17–26%) and dominant genetic (24%; 95% CI 17–31%) factors. Environmental influences (55%; 95% CI 51–60%) explained the remaining variance. Significant resemblance between spouses was observed, which was best explained by phenotypic assortative mating, but it had only a small effect on the genetic variance (1% of the total variance). There was no effect of cultural transmission from parents to offspring.

## Introduction

Borderline personality disorder (BPD) is characterized by emotional lability, impulsivity, interpersonal difficulties, identity disturbances, and cognitive impairments [1]. BPD is associated with a number of negative outcomes, including suicidal behavior, frequent emergency room admissions, substance abuse, impaired occupational functioning, and poor quality of interpersonal relationships. Individuals with BPD are well-represented in treatment settings, accounting for 10% of all outpatients and 15–20% of all inpatients [2]. Recent estimates from general population of the United States suggest that approximately 1% of adults meet diagnostic criteria for this disorder. BPD is equally prevalent among men and women and more likely to be diagnosed in early adulthood [3].

To date, genetic research on individual differences in BPD has been limited to non-twin family studies and classical twin studies. Family studies have consistently shown increased rates of BPD in family members of BPD patients [4]–[6], and twin studies of BPD reported heritability estimates around 40% [7]–[9]. Classical twin studies are important to detect whether there are genetic influences on BPD features. By including siblings, spouses and parents of twins in the study several additional research questions can be answered.

Firstly, adding data from siblings to the classical twin model results in a considerable increase in power to detect non-additive genetic effects [10]. Non-additive genetic effects can consist of interactions between alleles within a locus (dominance) or across different loci (epistasis). In this study, non-additive genetic effects are modelled as dominance. Using extended twin family designs, dominant genetic effects have been detected for many personality traits [11]–[15]. Lake et al. [16], for example, examined individual differences for neuroticism in 45,850 members of extended families from Australia and the United States, and found that additive genetic effects explained 28 to 36% of the variation and dominant genetic effects explained 13 to 17% of the variation. Neuroticism is suggested to be at the core of many features of BPD (e.g. negative emotionality, sensitivity to stress)[17] and empirical studies have found strong associations between BPD and neuroticism [18], [19]. We therefore hypothesize that dominant genetic effects may also influence BPD features.

Secondly, the effect of assortative mating, meaning that spouses are more similar for a trait or disorder than expected under random mating [20], [21], can be detected and accounted for by including data from parents and spouses of twins. Some degree of assortative mating is often found for psychiatric disorders and related phenotypic traits. For depressive disorders, a meta-analysis reported marital resemblance for depression in twelve of seventeen studies [22]. Studies on the etiology of spousal similarity for psychiatric disorders were carried out by Maes et al. [23] and van Grootheest et al. [24] in population-based samples. Several psychiatric diagnoses were examined, including generalized anxiety disorder, major depressive disorder, obsessive compulsive disorder, panic disorder and phobias. Moderate spousal correlations were seen for most psychiatric diagnoses. Social homogamy, marital interaction and phenotypic assortment are possible explanations for spousal similarity. Social homogamy refers to the tendency of spouses to have similar social backgrounds. Marital interaction means that spouses living together experience mutual influences which make them resemble each other, or that there are active influences of one spouse's phenotype on the other spouse's phenotype. Phenotypic assortment refers to the tendency of individuals to select their partner based on the partner's phenotype. The three mechanisms for spousal similarity have different implications for genetic analysis. Data of spouses of monozygotic and dizygotic twins provide information on which mechanism of assortment is most likely and should be included in the genetic analyses [23]–[26].

Although the classical twin design offers information about the influence of shared environment, it is not informative about how much of the shared environment is transmitted from parents to offspring. By adding phenotypic data from parents to the classical twin design vertical cultural transmission, reflecting the non-genetic influence of the parents' BPD features on their offspring, can be tested. Because BPD features have a heritable component [7] vertical cultural transmission will lead to genotype-environment correlation [27], [28].

In this study, we examine the genetic and environmental influences on individual differences in BPD features using an extended twin-family design. We collected data on BPD in twins, their spouses, siblings and parents. Analyzing the data from family members simultaneously in one model allows for testing of additive and dominant genetic effects, individual specific environmental influence, assortment and cultural transmission [29], [30].

## Methods

### Participants

Twins and their parents, siblings and spouses registered with the Netherlands Twin Register [31] and the East Flanders Prospective Twin Survey [32] were approached by mail and invited to participate in the study by completing a questionnaire. The total sample for analysis consisted of 5,017 twins and 1,266 siblings, 3,064 parents and 939 spouses of twins from 4,015 families. An overview of the sample characteristics is given in Table 1. Zygosity of 3,282 same sex twins was determined either from DNA typing (*N* = 1,907) or from self-report answers to eight survey questions on physical twin resemblance and confusion of the twins by family members and strangers. Based on the answers to these items from all longitudinal surveys, zygosity was assigned. A total of 1,045 twins were of opposite sex and therefore classified as dizygotic. Agreement between zygosity based on survey questions and zygosity based on DNA typing was 97% [33]. Details on response rates, demographic characteristics and zygosity procedures can be found elsewhere [7], [34], [35]. The study was approved by the Central Ethics Committee on Research involving human subjects of the VU University Medical Center, Amsterdam, an Institutional Review Board certified by the US Office of Human Research Protections (IRB number IRB-2991 under Federal wide Assurance-3703; IRB/institute codes, NTR 03-180). All subjects provided written informed consent.

**Table 1:** Number of twins, siblings, parents and spouses and their mean age (standard deviation) and age range.

	N	Mean age (SD)	Age range
*Twins*			
Monozygotic males	757		
Dizygotic males	389		
Monozygotic females	1,894		
Dizygotic females	932		
Dizygotic opposite sex males	417		
Dizygotic opposite sex females	628		
Total	5,017	33.7 (11.0)	18–86
*Siblings*			
Brother	472		
Sister	794		
Total	1,266	38.1 (12.3)	18–90
*Parents*			
Fathers	1,357		
Mothers	1,707		
Total	3,064	57.5 (6.5)	34–87
*Spouses*			
Male spouses	595		
Female spouses	344		
Total	939	38.0 (12.2)	19–80

### Measures

BPD features were measured by a Dutch translation of the 24-item *Personality Assessment Inventory-Borderline Features scale* (PAI-BOR) [36], [37]. The PAI-BOR consists of 24 items that are rated on a four-point scale (0 to 3; *false, slightly true, mainly true, very true*). The items consist of statements concerning, for example, stability of mood and affects, emotionally responsiveness, anger control, self image, feelings of emptiness, intense and unstable relationships, loneliness, impulsivity, self harm and recklessness. Several studies have supported the reliability and the validity of PAI-BOR scores in indexing the degree to which BPD features are present [36], [38]–[41]. Receiver operating characteristic analyses showed that the PAI-BOR discriminates well between BPD patients and patients with major depression disorder or dysthimia (area under the curve = 0.78). When interpreting the continuous PAI-BOR score as a categorical measure of BPD, at the best cut-off point of a score of 42, the sensitivity (proportion of individuals correctly classified as BPD) was 71% and the specificity 69% (1-specificity reflects the proportion of individuals falsely classified as BPD) [42]. Multigroup confirmatory factor analysis showed that the PAI-BOR is measurement invariant across sex and age [43]. The test-retest reliability and internal consistency (Cronbach's α) of the Dutch version of the PAI-BOR are 0.78 and 0.84, respectively [7]. The PAI-BOR was scored according to the manual, which states that at least 80% of the items must be answered to calculate a sum score and that missing and ambiguous answers should be substituted by a zero score [36].

### Genetic modelling

The classical twin design makes use of the different genetic relatedness of monozygotic (MZ) and dizygotic (DZ) twins to disentangle genetic and environmental influences on the variance in a trait. MZ twins are genetically (nearly) identical while DZ twins share on average 50% of their segregating genes, like non-twin siblings. The more similar MZ twins are relative to DZ twins, the more variability in a trait is caused by genetic effects. When there is no difference in resemblance between MZ and DZ twins, shared environmental influences are most likely the cause of the resemblance between twins. Genetic effects can act in an additive (A) or non-additive, or dominant (D; dominance) manner. Environmental effects can be common to members of the same family (C) or unique to an individual (E).

Adding data from siblings, spouses and parents of twins to the classical twin study has several advantages. Firstly, it provides the information and statistical power to distinguish between A and D, which is poorly achieved with the classic twin design [44], [45].

Secondly, the effects of assortative mating can be examined. In the classical twin design these may be confounded with the effects of the shared environment [46]. Information on the process of assortment (phenotypic assortment, marital interaction or social homogamy) can be deduced from the MZ and DZ co-twin spouse correlations. By comparing these correlations, a distinction can be made between phenotypic assortment and social homogamy. If assortment is primarily based on phenotypic assortment, the correlation between an MZ twin and their co-twins' spouse must be higher than the correlation between a DZ twin and their co-twins' spouse [25], [47]. If the trait is heritable, assortative mating increases genetic variance in the offspring generation because genetic effects in the parental generation are correlated. The correlation between the genotypes of parents will also increase the resemblance between parents and their offspring and among siblings [48]. When assortative mating for a heritable trait is not explicitly modelled, heritability estimates may become biased. For example, in the classical twin study, heritability estimates will be biased downwards and spurious evidence for shared environment may be found [23]. If assortment results from marital interaction, the spouse correlation increases as a function of duration of marriage and in general the correlation between parents of twins will be higher than between twins and their spouses [24].

Thirdly, including parents of twins into a study can provide information about cultural transmission from parents to offspring. Cultural transmission increases the parent-offspring correlation as well as the correlation among their offspring. In the classical twin design, cultural transmission will be accounted for as C. In an extended twin design cultural transmission can be distinguished from other forms of C, assuming that vertical cultural transmission from parents to offspring is based on the measured phenotype of the parents [46]. Factors that contribute to cultural transmission may be ‘taught’ from parents to their offspring in the form of imitation, customs or preferences, and have direct effects on behavioural phenotypes through processes of social learning or modelling. In contrast, non-transmittable shared-environment comprises environmental conditions shared by relatives reared together within a generation [49]. Importantly, if parents transmit both genes and environment, this induces a gene-environment correlation, as a consequence of the contribution of the parental phenotype, which is partly genetic in origin, to the offspring's environment [46].

### Resemblance among relatives

**Figure 1:**
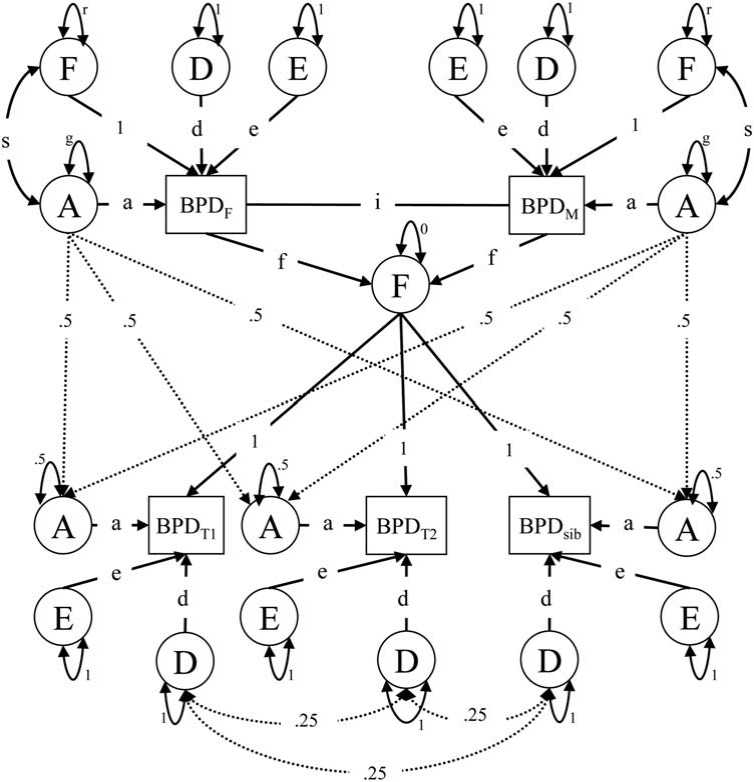
Family resemblance model for twins (BPD_T1_ and BPD_T2_), siblings (BPD_sib_) and parents (father, BPD_F_; mother, BPD_M_). A additive genetic variance, *a* factor loading of A, D dominant genetic variance, *d* factor loading of D, E unique environmental variance, *e* factor loading of E, F vertical cultural transmission, *f* factor loading of F, *g* additive genetic variance, *r*, variance due to cultural transmission, *s* genotype environment correlation (*g*, *r* and *s* are constrained as a function of offspring generation parameters), *i* assortment. For clarity reasons only one non-twin sibling is drawn, although more are used in the analyses.

In a first step, the resemblances between pairs of family members with different degrees of genetic relatedness were summarized by correlations. Correlations were estimated conditional on sex, for MZ and DZ twins, parent and offspring, sibling pairs, and for spouses (parents of twins and twins with their spouse). Simultaneously, means, variances and regression of BPD scores on age and sex were estimated. We tested for differences in correlations between DZ twins and siblings, for sex effects on twin and parent-offspring correlations and for regression effects of sex and age on the PAI-BOR scores. Next, the contribution of genetic and environmental factors to the variation in BPD features was estimated. Genetic modelling of the data was based on a re-parameterization of the model proposed by Fulker [30], of mixed genetic and cultural transmission described by Neale and colleagues [50]. The analysis of a univariate phenotype does not provide sufficient information to estimate the contribution of dominance, cultural transmission and shared environment. Based on the correlation structure of the data and prior analyses [7] we assumed that C beyond cultural transmission did not contribute to the variance in BPD features. Figure 1 presents the path diagram of a model in which the phenotypic variance is explained by additive (A) and dominant (D) genetic variation, unique environmental variation (E), vertical cultural transmission (F) and genotype- environment covariance (*s*). The use of parental data entails the assumption that assortative mating, genetic and cultural transmission and gene-environment correlation remain constant from generation to generation [25]. Therefore, the parameters *g* (genetic variance), *r* (variance due to vertical cultural transmission) and *s* (gene-environment covariance) in the parental generation are constrained in the model fitting as a function of the parameters in the offspring generation.

The additive genetic variance is perfectly correlated in MZ twins. For DZ twins and siblings the correlation between the latent A factors is 0.5. These coefficients are based on the assumption of random mating in the population [48]. They imply that, if h^2^ is the heritability of a trait, the correlation (due to A) between parents and offspring and between siblings equals ½h^2^. Under assortative mating, there is an increase in the genetic variance, which will increase the resemblance between parents and offspring as well as between siblings, i.e. *r*_g_>0.5 [51]. The effect of phenotypic assortment is included in the model as represented by the co-path *i.* The copath represents an extrinsic correlation that influences the covariance structure of the spouses' latent variables but does not contribute to their variance [52]. Dominant genetic variation results from the interaction or combination of alleles at a particular locus. Offspring receive only one allele from each parent and not a combination of two alleles, thus assuming outbred mating the chance that two siblings receive the same allele is 0.5×0.5 resulting in a correlation of 0.25 between the latent D factor for DZ twins and a correlation of zero between parents and offspring. Variance due to D is not expected to change as a product of assortative mating, since BPD characteristics are assumed to be influenced by a large number of genes [48], [51].

### Model fitting 

Several models of familial resemblance were fitted to the data. We first estimated correlations between relatives and then fitted a series of genetic models to the data. In the first model (model I), A, D, E, cultural transmission and resulting genotype environment correlation are specified. Model II tests the significance of cultural transmission and genotype environment correlation, model III the significance of D and model V the significance of assortment. Finally, model IV tests the significance of A. Because the data showed a somewhat skewed distribution with a tail to the right, a square root transformation was applied. All analyses were performed in the software package Mx [53], using the raw-data full-information maximum-likelihood approach. The fit of the different models was evaluated by means of hierarchical log-likelihood ratio test (LRT) to select the simplest model that best explains the data among a set of possible models. The difference between the negative log likelihood (-2LL) of the two models has a χ^2^ distribution and the degrees of freedom (df) for this test equals the difference in the number of estimated parameters in the two models. A non-significant *p*-value means that the constrained model is not significantly worse than the model and is kept as the most parsimonious and best fitting model. Because of the large sample size a *p*-value of 0.01 was chosen.

## Results

Table 2 gives the estimates for the intercept and regression coefficients for sex and age and estimates of the PAI-BOR score for 18 year old men. The sex and age regression coefficients represent the deviation per increasing age year and the deviation for women. The upper part of Table 3 shows the results of the tests on the regression coefficients and the variances. Both the age and sex regression coefficients on the mean PAI-BOR score were significant, with younger women showing most BPD features (both *p*<.001). The effects of sex and age on the PAI-BOR scores were therefore included in all genetic models as a regression coefficient. Variances were equal for men and women.

**Table 2:** Estimates for borderline personality intercept (estimated for men at age 18), regression coefficients for sex (deviation in women) and age (per year) from the regression equation and standard deviations for untransformed data and square root transformed data (estimates plus 95% confidence intervals).

	Untransformed data	Transformed data
Intercept	18.00 (17.24,17.77)	4.10 (4.03,4.17)
β_age_	−.07 (−.09,−.05)	−.008 (−.009,−.007)
β_sex_	1.57 (1.14,2.01)	.21 (.16,.25)
Standard deviation	8.02 (7.86,8.18)	1.00 (.99,1.01)

**Table 3:** Tests of variances, means and correlations.

Model	vs	-2LL	df	χ^2^	Δdf	*p*
1. Saturated model		26,025.096	9,329			
2. Variance males = variance females	1	26,025.149	9,330	0.053		.818
3. Sex effect on mean = 0	2	26,120.790	9,331	95.641	1	<.001
4. Age effect on mean = 0	2	26,155.259	9,331	130.110	1	<.001
5. *r*DZM = *r*Brother - brother = *r*DZF = *r*Sister - sister = *r*DOS = *r*Brother - sister	2	26,030.852	9,335	5.703	5	.336
6. *r*MZM = *r*MZF	5	26,031.040	9,336	0.188	1	.665
7. *r*Father - mother = 0	6	26,091.713	9,337	60.673	1	<.001
**8. ** ***r*** **Father - son = ** ***r*** **Father - daughter = ** ***r*** **Mother - son = ** ***r*** **Mother - daughter**	**6**	**26,041.683**	**9,339**	**10.643**	**3**	**.014**

Note: vs = versus, -2LL = -2 log likelihood, df = degrees of freedom, *p* = *p*-value

The best fitting model is printed in bold.

**Figure 2:**
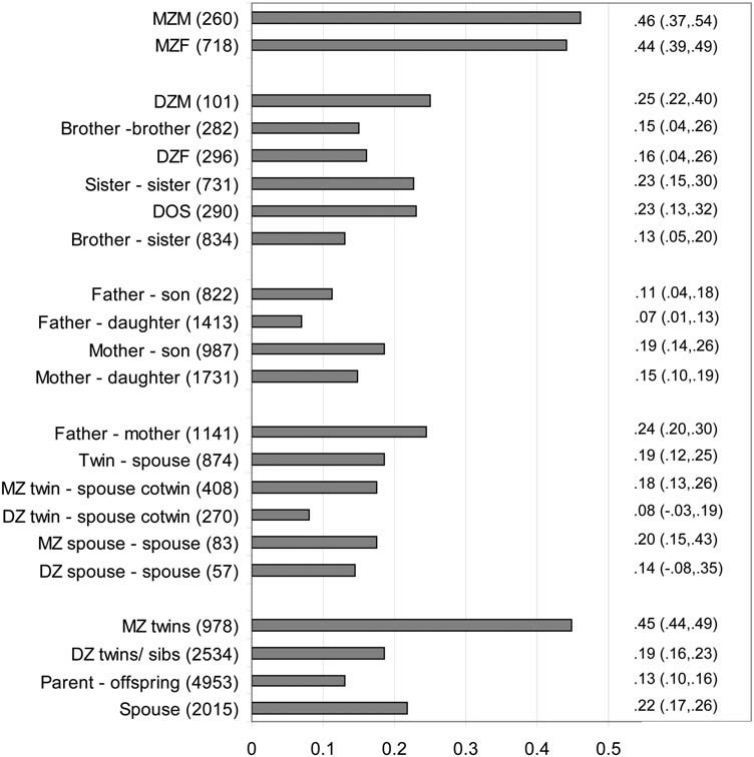
Correlations for BPD features between family members of different degrees of relatedness (number of pairs) and 95% confidence intervals. The bottom 4 bars collapse across categories above.

The bottom part of Table 3 shows the results of the tests on the correlations. There were no sex differences in twin and sibling correlations (all *p*>.01), indicating that there were no sex differences in the heritability of BPD features, the same genes influence BPD features in men and women (test not shown in Table 3) and there is no specific twin environment (all *p*>.01). The MZ twin correlation was .45 and the DZ/sib correlation was .19 suggesting that around 50% of the variance in BPD features can be attributed to genetic factors and that part of the genetic variance might be dominant. Resemblance between mothers and their offspring was equal to the resemblance between fathers and their offspring (*p* = .014). The parent-offspring correlation (*r* = .13) was somewhat lower than the DZ/sibling correlation which is consistent with the presence of dominance. There was a significant association between the PAI-BOR scores of twins and the score of their spouses (*r* = .19). The correlation between MZ twins and their co-twins spouse (*r* = .18) was higher than the correlation between DZ twins and their co-twins' spouse (*r* = .08) which suggests that non random mating is primarily based on phenotypic assortment. The spouse correlation in the parental generation was .24 indicating that in addition to phenotypic assortment, there may be some influence of marital interaction. The estimates for the familial correlations for pairs of family members with different degrees of genetic relatedness are summarized in Figure 2.

### Genetic modelling

Table 4 shows the result of genetic model fitting. Model I specifies effects of A, D and E, assortment and cultural transmission. The model is just identified, meaning that the number of free parameters in the model equals the number of peaces of information, and provides the same fit to the data as the correlation model (model 8) in Table 3. From the estimates for the path coefficients, the influence of A on individual differences in BPD features can be obtained by the product of the additive genetic path coefficient squared and the additive genetic variance divided by the total variance (A = *a* ^2^ * *g*/total variance). The influence of assortment on A can be calculated by A - a^2^ showing that in this model 3.0% of the additive genetic variance (38.5%) is explained by assortment. Non additive genetic effects (*d* ^2^/total variance) explained 11.4% of the variance. Unique environmental effects (*e* ^2^/total variance) explained 55.3% and negative cultural transmission (*r*) explained 1.3% of the variance. Genotype-environment covariance (as*sa/total variance) was estimated to be negative, resulting in a negative contribution of 6.4% of the variance in BPD features. In model II (dominance model without cultural transmission), additive genetic effects explained 21.3% (1.1% due to assortment) and dominant genetic effects explained 23.9% of the variance in BPD features. The remaining variance was accounted for by unique environmental influences. The fit of model II is not significantly worse than the fit of model I (χ^2^_(1)_ = .50, *p* = .480) which indicates that there is no significant effect of cultural transmission and resulting genotype environment correlation. Comparing the fit of model II with the fit of model III shows that removing D from the model results in a significant deterioration in the fit of the model (χ^2^_(1)_ = 47.0,*p*<.001). Model IV (versus model III), shows that the influence of A is highly significant since removing it from the model results in a considerable worsening of fit (χ^2^_(1)_ = 293.2, *p*<.001). Finally, comparing model V with model II shows that there is a significant effect of assortment (χ^2^_(1)_ = 62.0, *p*<.001). Comparing the fit of the different models showed that the ADE model best explained the data.

**Table 4:** Maximum likelihood parameter estimates and goodness of fit indices from the extended twin design for borderline personality (95% confidence intervals in parentheses for the best fitting model).

	I	II	III	IV	V
Additive genetic path (*a*)	.593	**.447 (.39, .50)**	.545	-	.465
Dominant genetic path (*d*)	.336	**.487 (.42, .54)**	-	-	.480
Specific environment path (*e*)	.741	**.738 (.71, .76)**	.820	.996	.738
Assortment (*i*)	.251	**.251 (.21, .30)**	.240	.246	-
Additive genetic variance (*g*)	1.088	**1.056 (1.04, 1.07)**	1.084	1.000	1.000
Variance due to cultural transmission (*r*)	.013	**-**	-	-	-
A-C covariance (*s*)	−.054	**-**	-	-	-
Cultural transmission (*f*)	−.073	**-**	-	-	-
-2 LL	26,041.683	**26,042.184**	26,089.204	26,382.414	26,104.180
Degrees of freedom	9,339	**9,340**	9,341	9,342	9,341
χ^2^	-	**.501**	47.521	340.731	62.497
Δ degrees of freedom	-	**1**	2	3	2
*p*	-	**.480**	<.001	<.001	<.001

Model I: cultural transmission model

Model II: dominance model; no cultural transmission

Model III: as model II, no dominance

Model IV: as model III, no additive genetic effects

Model V: as model II, no assortment

Best fitting model printed in bold.

## Discussion

This is the first study that analyzes borderline personality data from twins and their family members simultaneously providing a powerful design to distinguish between additive and dominant genetic effects and to detect non-random mating, cultural transmission and genotype-environment correlation. A genetic model in which additive genetic effects (21.3%; 95% CI 16%–26%), dominant genetic effects (23.9%; 95% CI 17%–31%) and unique environmental influences (54.9%; 95% CI 51%–60%) explained the variance in BPD features best explained the data. There was no evidence for shared environmental influences, which is a common finding for a range of personality traits and personality disorders. The effect of phenotypic assortment was included in the genetic model, but it had only a small effect on the genetic variance.

The presence of significant dominant genetic effects is in line with what is often suspected for personality traits, but not detected due to a lack of statistical power in relatively small twin studies. Our results showed that BPD features are genetic in origin but only partly transmitted from parents to offspring because dominant genetic effects influence borderline personality only in combination with other genes. These combinations are not shared by parents and offspring. Keller et al. [11] used a twin-sibling design to estimate genetic and environmental effects on Eysenck's and Cloninger's personality dimensions using data from over 12,000 twins and siblings. They found that 0 to 34% of the variance in these personality dimensions was explained by additive genetic effects and 11 to 35% was explained by dominant genetic effects.

The finding of dominance for personality traits is not uncommon, but there may be alternative explanations for these data. The parent-offspring correlation for BPD features was lower than the DZ/sibling correlation which is indicative of the presence of dominance but might also suggest genotype by age interaction, i.e. the expression of different genes at different ages or a change in genetic variance as a function of age. Gene by age interaction can inflate estimates of dominance because it will decrease the correlation between parents and offspring as a result of their differences in age. To investigate this alternative we first divided the twin sample into a group with roughly the same age as the parents in the total sample (N = 968, mean age 52.7 years) and a group with roughly the same age as the offspring in the sample (N = 4,047, mean age 29.1 years). The total variance did not differ between the two groups (χ^2^_(1)_ = .011, *p* = .916). The MZ and DZ twin correlations of the younger and older age groups were .472 versus .247, and .459 versus .095, suggesting that broad-sense heritability might be larger in the older generation. However, constraining the MZ and DZ twin correlations to be equal across age groups did not lead to a significant worsening of model fit (χ^2^_(1)_ = .051, *p* = .821 and χ^2^_(1)_ = 2.618,*p* = .106). Thus, heritability may not change as a function of age. Secondly, to investigate whether different genes are expressed at different ages, we selected a group of siblings less then 4 years (190 pairs) and a group of siblings 4 years or more apart in age (212 pairs). The PAI-BOR correlations for siblings in these groups were .208 and .327 and the resemblance between siblings thus does not decrease as the age difference between them increases. The correlations in the two sibling groups could be constrained to be equal (χ^2^_(1)_ = 1.69, *p* = .194). This suggests that the same genes influence BPD features at different ages.

The largest part of the variance in borderline personality was explained by unique environmental influences (54.9%). Several studies demonstrated that traumatic life events such as sexual and physical abuse, parental divorce or illness or parental psychopathology are important risk factors for the development of BPD [54]–[57]. The interaction, however, between the influences of genes and environment on the development of BPD has not been studied. Gene by environment interaction implies that genes determine the degree to which an individual is sensitive to an environment. In the presence of gene-environment interaction, individuals with a ‘sensitive’ genotype will be at greater risk of developing BPD if an undesirable environment is present, than individuals with an ‘insensitive’ genotype. In the present study, gene-environment interaction would be included as part the unique environmental variance. Future research should focus on possible sources of unique environmental effects and gene-environment interaction to develop a comprehensive model of the development of BPD.
